# Homocysteine enhances neural stem cell autophagy in in vivo and in vitro model of ischemic stroke

**DOI:** 10.1038/s41419-019-1798-4

**Published:** 2019-07-22

**Authors:** Mengying Wang, Xiaoshan Liang, Man Cheng, Liu Yang, Huan Liu, Xuan Wang, Na Sai, Xumei Zhang

**Affiliations:** 1 Department of Nutrition and Food Science, School of Public Health, Tianjin Medical University, Tianjin, China; 2 Tianjin Key Laboratory of Environment, Nutrition and Public Health, Tianjin, China

**Keywords:** Neural stem cells, Brain injuries

## Abstract

The elevated level of the amino acid metabolite homocysteine (Hcy) is known as a risk factor for ischemic stroke. The molecular mechanisms responsible for neurotoxicity of Hcy remain largely unknown in ischemic brains. The previous studies have shown that Hcy decreases the proliferation and viability of neural stem cells (NSCs) in vivo and in vitro. Autophagy is required for the maintenance of NSCs homeostasis. In the current study, we hypothesized that the toxic effect of Hcy on NSCs may involve the changes in autophagy level following cerebral ischemia/reperfusion injury. The results showed that Hcy reduced cell viability, increased LDH release, and induced nonapoptotic cell death in primary NSCs exposed to oxygen–glucose deprivation)/reoxygenation (OGD/R). Treatment with autophagy inhibitor 3-methyladenine (3MA) partly reversed the decrease in the viability and prevented LDH release triggered by Hcy combined with OGD/R. Increased punctate LC3 dots co-localizing with Nestin-stained NSCs were also observed in the subventricular zone of Hcy-treated MCAO animals, which were partially blocked by 3MA. In vitro studies further revealed that Hcy induced the formation of autophagosomes, markedly increased the expression of the autophagic markers and decreased p-ERK, p-PI3K, p-AKT, and p-mTOR levels. In addition, MHY1485, an activator of mTOR, reduced Hcy-induced increase in LC3 and Beclin 1 protein levels, meanwhile ERK and PI3K activators (TPA, curcumin for ERK and IGF-1 for PI3K, respectively) enhanced Hcy-triggered mTOR inhibition in OGD/R NSCs. Our findings suggest that Hcy may cause excessive autophagy by downregulation of both PI3K-AKT- and ERK- dependent mTOR signaling, thereby facilitates the toxicity of Hcy on NSCs in ischemic brains.

## Introduction

Ischemic stroke, as a major pathological stroke type, is one of the leading causes of mortality and disability worldwide. Ischemic insults have been reported to trigger neurogenesis from neural stem cells (NSCs)/progenitor cells located in the subventricular zone (SVZ) and the subgranular zone (SGZ) in the dentate gyrus of adult mammalian brains, and these cells migrate to the boundary of the ischemic lesion which has been proposed to aid repair of the injured network in ischemic brains^1^. Therefore, any environmental damage to NSCs survival may result in the inability to replace lost or injured neurons and could be a contributing factor to the brain atrophy. It is thus critical to understand the role of the microenvironment in the fate of adult NSCs.

Homocysteine (Hcy), a sulfur-containing amino acid, was derived from the methionine metabolism. Hcy accumulation in peripheral blood could induce hyperhomocysteinemia (HHcy), which is known as an independent risk factor for some central nervous system diseases such as Alzheimer’s disease, vascular dementia, cognitive impairment, and stroke^2–5^. Previous studies showed that neural cells are sensitive to prolonged HHcy treatment, and thus it contributes to chronic progressive neurological disease^6,7^. In addition, the neurotoxicity of Hcy involves negative regulation of NSCs viability, proliferation, and differentiation capacities^8,9^. For instance, Rabaneda et al. presented in vitro and in vivo evidence demonstrating that Hcy exerted an inhibitory effect on mice adult brain neurogenesis and antiproliferative effect on basic fibroblast growth factor (bFGF)-stimulated neural progenitor cells isolated from the postnatal SVZ^10^. However, the underlying mechanism for direct neurotoxic effects of Hcy on NSCs has remained obscure.

Autophagy is a regulated process of degradation and recycling of cellular constituents that occurs in response to various cellular stresses and/or extrinsic stimuli such as starvation, hypoxia, drugs, infection, and growth factor deprivation^11,12^. Emerging evidence indicates that autophagy was involved in modulation of the embryonic neurogenesis as well as the injury repair of adult brain^13,14^. However, little is known regarding the role of autophagy in the neurogenic response to external stimuli. He et al. showed that physical exercise such as voluntary running stimulated neurogenesis by enhancing proliferation and survival and can also induce autophagy in the brain^15^. In this study, we tested whether Hcy, as one form of environmental stimuli, affected autophagy in NSCs in in vitro and in vivo experimental cerebral stroke models.

It has been observed in several different cell types that Hcy-induced cell injury by stimulating autophagy. For instance, human umbilical vein endothelial cells damaged by Hcy exhibit features of autophagic/lysosomal cell death accompanying with the higher expression levels of the autophagy-related genes *Atg* 5 and *Beclin* 1, greater degradation of LC3 I to LC3 II and the cytoplasmic autophagic vacuoles^16^. Zhao et al. reported that Hcy treatment aggravated neuronal cell death, significantly increased the formation of autophagosomes and the expression of LC3B and Beclin 1 in the brain cortex after middle cerebral artery occlusion-reperfusion^17^. Yang et al. demonstrated that Hcy induces autophagy of hepatic cells both in vivo and in vitro^18^. Thus, it is possible that Hcy causes the decreased viability of NSCs via autophagy regulation.

Mammalian target of rapamycin (mTOR), a serine/threonine kinase, is a master regulator of autophagy^19–21^. The class I phosphoinositide-3 kinase (PI3K) and extracellular signal-regulated kinase (ERK) are two upstream targets of mTOR and positively regulate the mTOR activation^21^. Meanwhile, the Ser/Thr protein kinase AKT (protein kinase B) is a downstream target of PI3K and positively regulates proliferation, survival, cell size, cell migration, differentiation, and apoptosis^19^. Both the PI3K-Akt- and ERK-dependent mTOR signaling pathways are essential for neurogenesis from NSCs, as well as for regulation of autophagy^14^. In addition, it was suggested that oxygen–glucose deprivation (OGD) strongly induced autophagy in adult rat hippocampal NSCs via the increase of autophagy activity markers and the promotion of autophagosomes and autophagolysosome formation^5^. Thus we concluded that Hcy may further regulate the autophagy level raised by ischemia/reperfusion injury in NSCs through PI3K-Akt- and ERK-dependent mTOR signaling pathways.

The present study was designed to elucidate the role of autophagy in the toxic effect of Hcy on NSCs using the OGD/R cell model as well as brain ischemia/reperfusion model. Moreover, the potential for PI3K-Akt- and ERK- dependent mTOR pathways was investigated during the joint regulation of Hcy and OGD/R on autophagic activity.

## Results

### Suppression of autophagy attenuates the effect of Hcy on OGD/R NSCs viability

To verify the toxic effect of Hcy on the cultured rat NSCs subjected to OGD/R, the cell viability was determined by the MTT assay, and cell necrosis was evaluated by the LDH-release assay. After exposure to OGD 1% O_2_, 5% CO_2_, and 94% N_2_ for 1 h, followed by an additional 24 h under normoxic conditions, the cell viability of OGD/R group was significantly decreased, and LDH release was greatly increased, when compared with the normal group (*P* < 0.05). Exposure of OGD/R NSCs to 300 μM Hcy, for six consecutive days, resulted in a strong decrease in cell viability and a further increase in LDH release, compared with that in the OGD/R group (*P* < 0.05).

**Fig. 1:**
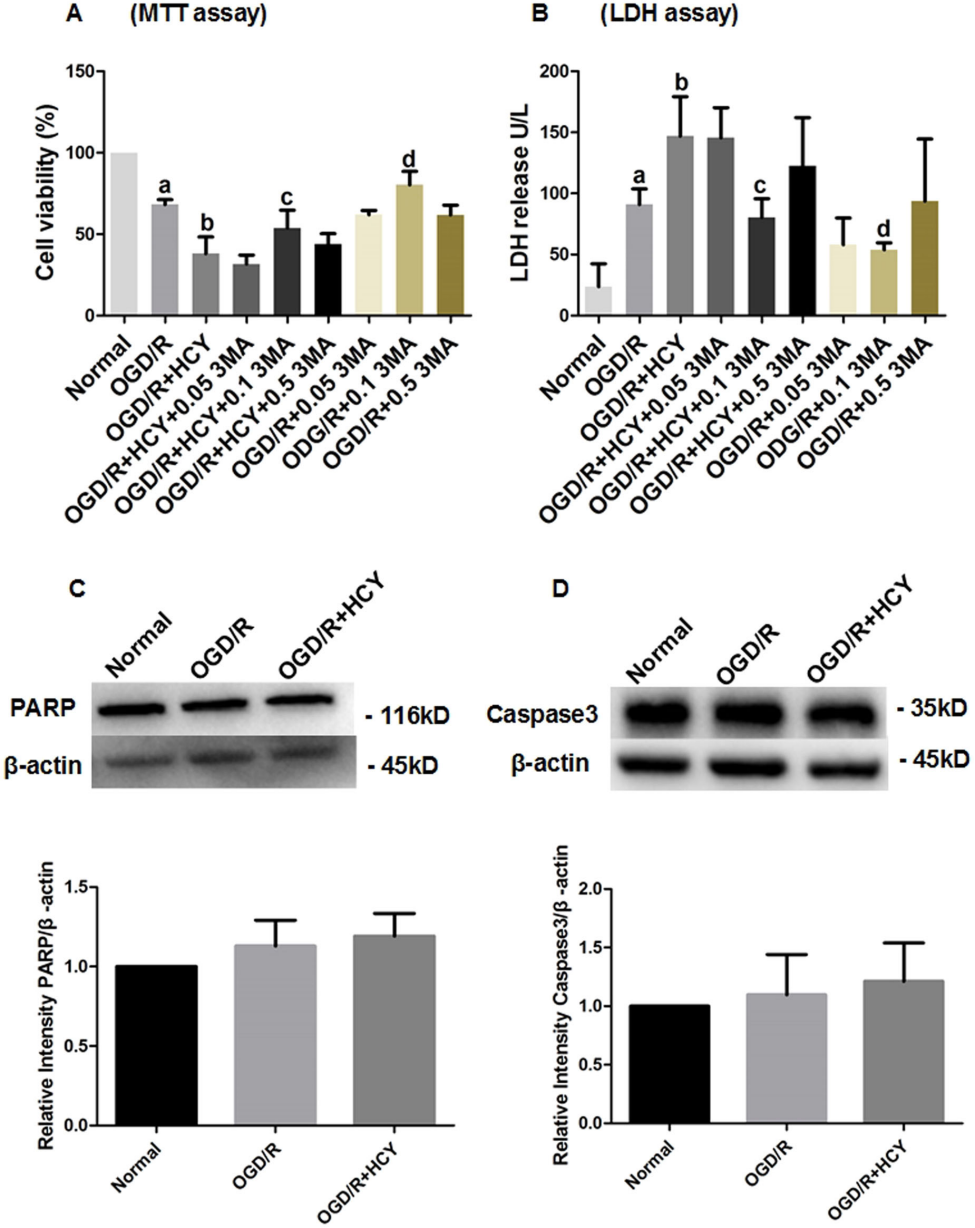
Effects of 3MA treatment on Hcy-induced cytotoxicity in OGD/R NSCs. **a** After exposure to 3MA at three different concentrations (0.05, 0.1, or 0.5 mM) for 72 h, the cell viability was detected by the MTT assay. **b** Cell necrosis was evaluated by the measurement of LDH in media. 0.05, 0.1, and 0.5 mM 3MA was used in the OGD/R + HCY + 0.05 3MA, OGD/R + HCY + 0.1 3MA, and OGD/R + HCY + 0.5 3MA groups, respectively. The data are average of three independent experiments. Note that the percentage of cell viability of other groups is normalized to the normal group. Data are expressed as mean ± SD. **a** *P* < 0.05 compared with the normal group. **b**, **d** *P* < 0.05 compared with the OGD/R group. **c** *P* < 0.05 compared with the OGD/R + HCY group. **c** Representative western blot for PARP. Quantification of PARP/β-actin graphed below. **d** Representative western blot for caspase 3. Bar graphs show semiquantitative levels of caspase 3 as determined by band density analysis. Data from three independent experiments. Note that the quantification of PARP or caspase 3 of other groups is normalized to the normal group. Data are expressed as mean ± SD. *P* > 0.05

3-Methyladenine (3MA) is an autophagy inhibitor and has been widely used as a pharmacological tool in the autophagy studies. To assess the role of autophagy in Hcy-treated NSCs subjected to OGD/R, the cells were treated with three different concentrations (0.05, 0.1, and 0.5 mM) of 3MA for 72 h, subsequently, cell viability and injury were evaluated by the MTT assay and LDH release. The results demonstrated that co-treatment Hcy with 0.1 mM 3MA in the OGD/R NSCs significantly promoted cell viability (Fig. 1a) and inhibited LDH release (Fig. 1b) (*P* < 0.05), compared with that in the OGD/R + HCY group. However, the effects of Hcy on cell viability and LDH release were not obviously blocked in the cells treated with 0.05 or 0.5 mM 3MA compared with the 3MA-untreated cells subjected to OGD/R. Taken together, these findings demonstrate that autophagy inhibition by 0.1 mM 3MA significantly abrogated Hcy-triggered suppression of cell viability and increase in cell necrosis in OGD/R-exposed NSCs.

To examine whether neural stem cell death induced by the OGD/R and Hcy treatment shows any features of apoptosis, the expression levels of the apoptosis-related proteins PARP and active caspase-3 were detected by western blotting, our results showed that there was no significant effect of Hcy on expression of these two proteins (Fig. 1c, d). Thus NSCs may undergo a caspase-3-independent, autophagic cell death following Hcy treatment.

### Hcy increases the protein levels of LC3 and Beclin 1 and promotes autophagosome formation in OGD/R-treated NSCs

**Fig. 2:**
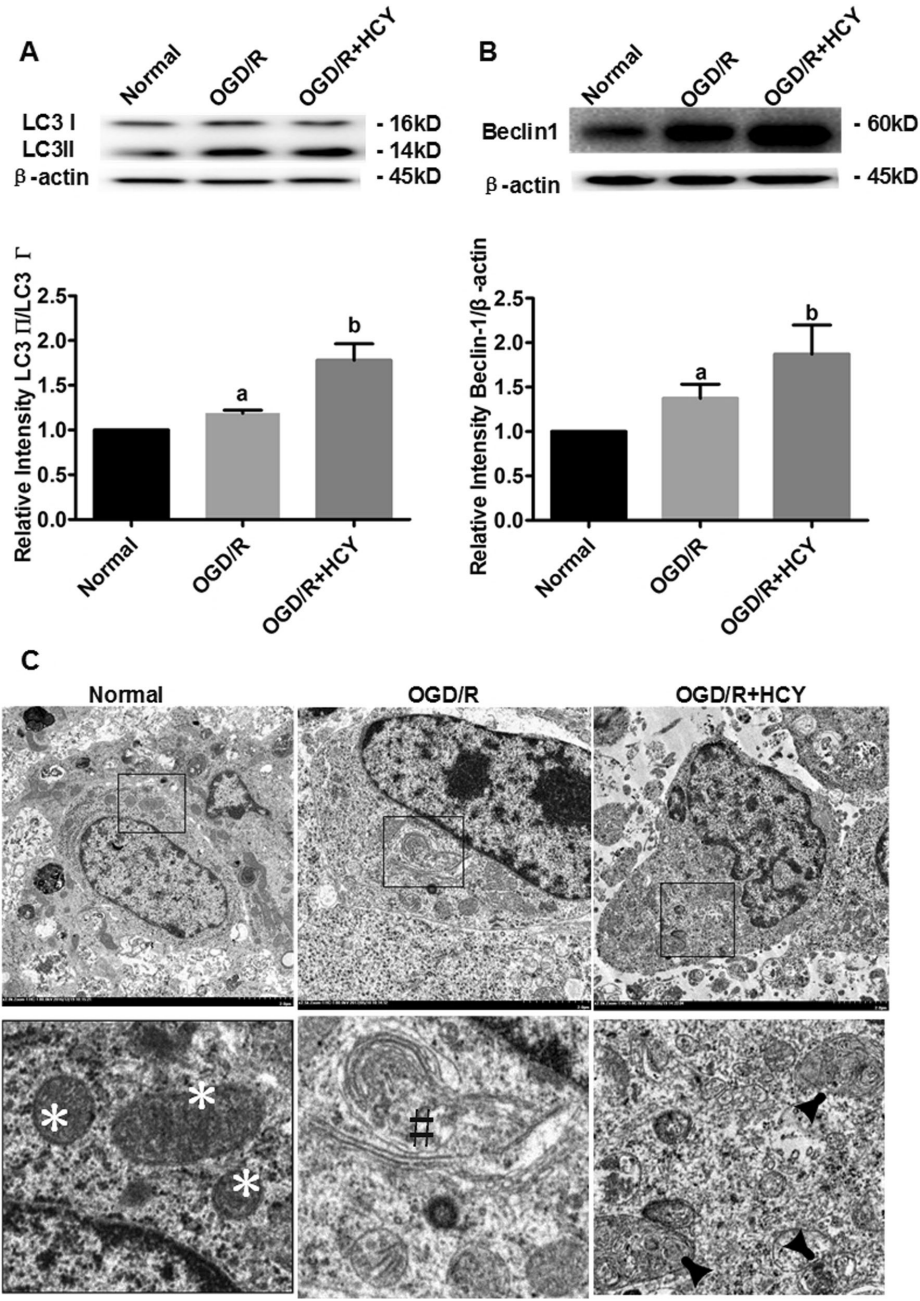
LC3 and Beclin 1 expression, and ultrastructure of autophagy in Hcy-treated NSCs exposed to OGD/R. **a** Representative western blot for LC3. Quantification of LC3 II/LC3 I graphed below. **b** Representative western blot for Beclin 1. Bar graphs show semiquantitative levels of Beclin 1 as determined by band density analysis. Data from three independent experiments. Note that the quantification of Beclin 1 or LC3 II/LC3 I ratio of other groups is normalized to the normal group. Data are expressed as mean ± SD. **a** *P* < 0.05 compared with the normal group. **b** *P* < 0.05 compared with the OGD/R group. **c** Representative electron photomicrographs of autophagosomes. Three panels in the bottom show the magnified images of the insets. Normal morphology of cytoplasm was indicated by the asterisk symbol. The autophagy formation was indicated by the hash symbol in the OGD/R group. The arrowheads show characteristic autophagosomes in the OGD/R + HCY group. Scale bar = 2 μm

Western blot analyses of LC3 (especially LC3 II/LC3 I ratio) and Beclin 1 were used to detect the generation of autophagosomes and the initiation of macroautophagic flux, respectively (Fig. 2a, b). Quantitative analysis revealed that OGD/R-treated NSCs exhibited the higher ratio of LC3 II to LC3 I and the raised protein level of Beclin 1, compared with the nontreated cells (*P* < 0.05). In addition, LC3 II/LC3 I ratio and Beclin 1 protein level were further upregulated by Hcy treatment in OGD/R NSCs, compared with that in the OGD/R group (*P* < 0.05).

The autophagosome formation was confirmed by transmission electron microscopy (TEM) after Hcy exposure. As shown in Fig. 2c, mitochondria had a dense matrix, neatly aligned cristae, and no signs of autophagy were detected in the normal group. In contrast, in the OGD/R group, the mitochondria were visibly swollen with partially broken or disorganized cristae, and the rough endoplasmic reticula developed cystic degeneration. A few double membrane-bound compartments that contained cytoplasmic material (autophagosomes) were visible. Damage of NSCs was more pronounced accompanied by more organelle lysis, and the augmented number of autophagy structures was observed in the OGD/R + HCY group than that in the OGD/R group. The data strongly suggested that overactivation of autophagy is triggered in the Hcy-treated NSCs exposed to OGD/R.

### Effect of Hcy on autophagy in NSCs in SVZ of ischemic brain

**Fig. 3:**
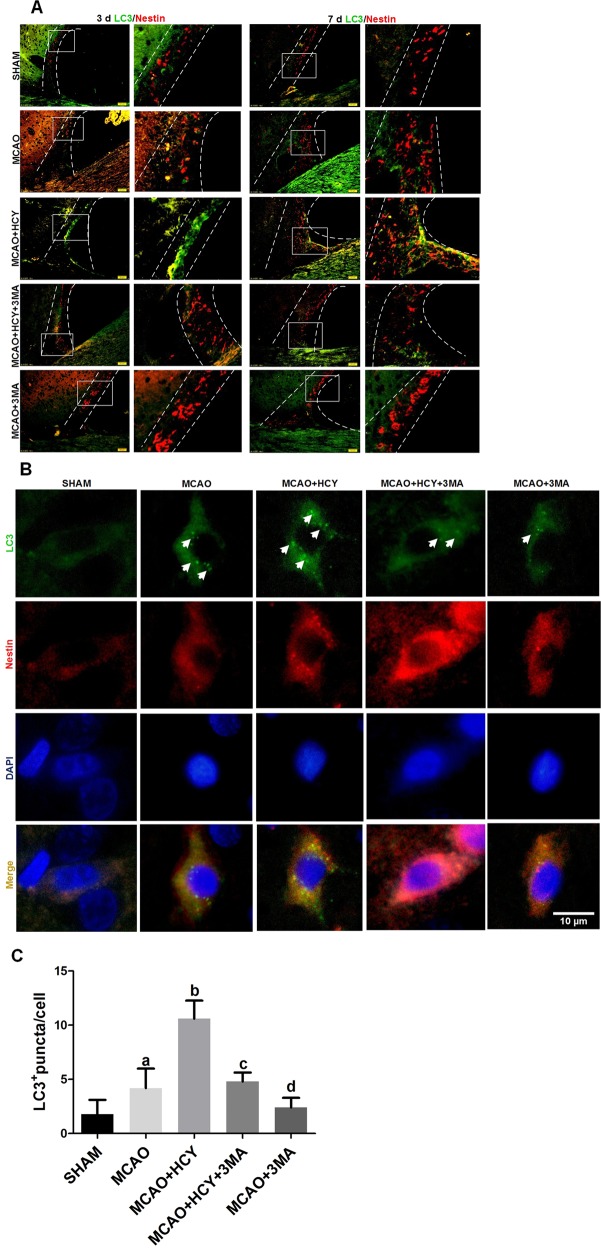
Hcy increases LC3 expression in the NSCs of SVZ after brain ischemia-reperfusion injury. **a** Representative images of double immunofluorescence staining for LC3 (green) and Nestin (red) in the SVZ for all experimental groups at 3 days (left panel) and 7 days (right penal) after operation, respectively. Scale bar = 50 μm. **b** Double immunofluorescent staining revealed accumulation of LC3 punctuated foci per cell. Green color, LC3 staining; red, Nestin staining; yellow, co-localization of LC3 and Nestin. Autophagosomes are recognized as punctate structures (white arrow). Scale bar = 10 μm. **c** The number of LC3 puncta mean ± SD per cell was counted and presented graphically. Data from three independent experiments. **a** *P* < 0.05 compared with the SHAM group. **b**, **d** *P* < 0.05 compared with the MCAO group. **c** *P* *<* 0.05 compared with the MCAO + HCY group

To identify further evidence supporting the role of autophagy in mammalian adult neurogenesis following Hcy treatment in vivo, the expression of autophagic protein LC3 was detected in NSCs of the SVZ (a key area for adult neurogenesis) in the adult rat brain after MCAO by double immunofluorescent staining. The results showed that LC3-positive cells were virtually absent in SHAM group, indicating that autophagy activity was very low. A moderate level of LC3 signal was detected in Nestin-positive NSCs 3 and 7 days after ischemic injury. A strong LC3 signal was detectable in NSCs of Hcy-exposed ischemic brains, whereas Hcy-induced increase in LC3 expression was prevented by 3MA treatment (Fig. 3a).

The above results were followed up by analysis for LC3 puncta (small green granules in Fig. 3b) per cell that indicate the conversion of LC3 (form I to II) and its recruitment into autophagosomes by immunofluorescence. As shown in Fig. 3b, in sham-operated animals, SVZ NSCs displayed diffuse and weak staining for LC3 in the cytosol. After ischemic injury, intense LC3 staining appeared granular in the cytosol of NSCs. A further increase in the number of punctated LC3 foci per cell was evident in the MCAO + HCY group, compared with that in the MCAO group, whereas application of autophagy inhibitor 3MA to ischemic rats attenuated significantly the level of LC3 puncta that was raised by Hcy (*P* *<* *0.05*, Fig. 3c). Obviously, 3MA partially antagonized Hcy-triggered autophagy in SVZ NSCs of the adult rat ischemic brain.

### Hcy induces autophagy via the mTOR signaling in OGD/R-treated NSCs

**Fig. 4:**
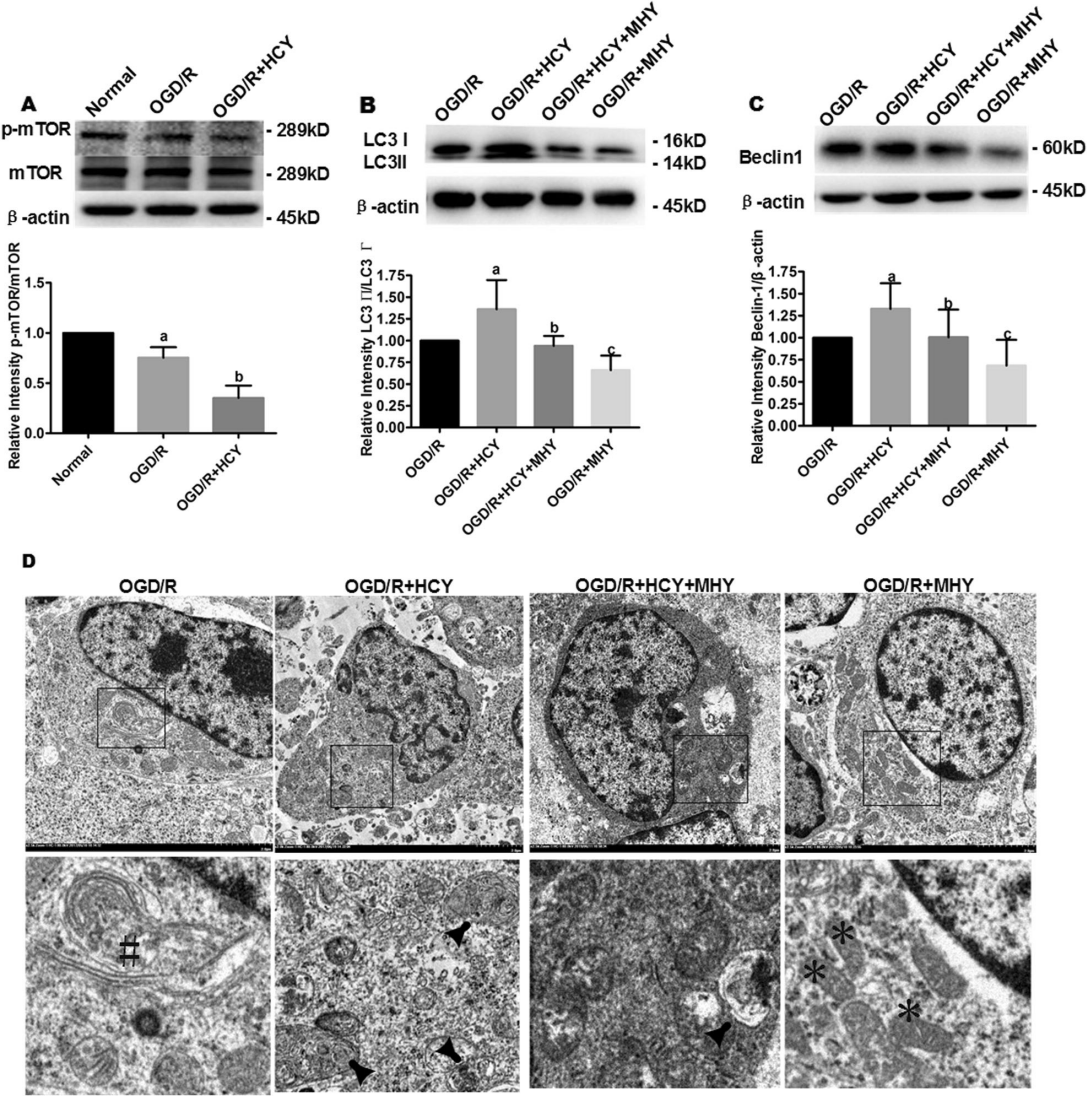
Hcy promotes autophagy in OGD/R NSCs by inhibiting the phosphorylation of mTOR. **a** Representative western blot for p-mTOR. Bar graphs show semiquantitative levels of p-mTOR as determined by band density analysis. Data from three independent experiments. Note that the quantification of p-mTOR of other groups is normalized to the normal group. Data are expressed as mean ± SD. **a** *P* < 0.05 compared with the normal group. **b** *P* < 0.05 compared with the OGD/R group. **b**, **c** Representative western blot for LC3 and Beclin 1. Bar graphs show semiquantitative levels of LC3 and Beclin 1 as determined by band density analysis, respectively. β-actin was used as a loading control. Data from three independent experiments. Note that the quantification of Beclin 1 or LC3 II/LC3 I of other groups is normalized to the OGD/R group. Data are expressed as mean ± SD. **a**, **c** *P* < 0.05 compared with the OGD/R group. **b** *P* < 0.05 compared with the OGD/R + HCY group. **d** Representative electron photomicrographs of autophagosomes. Four panels in the bottom show the magnified images of the insets. The autophagy formation was indicated by the hash symbol in the OGD/R group. The arrowheads show characteristic autophagosomes in OGD/R + HCY and OGD/R + HCY + MHY groups. Normal morphology of cytoplasm was indicated by the asterisk symbol. Scale bar = 2 μm

To determine the role of mTOR signaling (a critical negative regulator of autophagy) in Hcy-induced neurotoxicity, we investigated the expression of p-mTOR in NSCs after the combined exposure to Hcy and OGD/R by western blot. The results revealed that the phosphorylated mTOR protein level was significantly lower in the OGD/R group compared with the normal group (*P* < 0.05, Fig. 4a). The protein level of p-mTOR was further decreased in the OGD/R + HCY group compared with that in the OGD/R group (*P* *<* 0.05).

To further demonstrate that Hcy promoted mTOR-dependent autophagy in OGD/R-treated NSCs, MHY1485, an mTOR activator, was added to the culture medium in the OGD/R + HCY group. As shown in Fig. 4b, c, co-treatment with Hcy and 10 µM MHY1485 resulted in a significant decrease of LC3 II/LC3 I ratio and Beclin 1 expression. In addition, a more intact cellular structure and a decreased number of autophagosomes were also confirmed by TEM in the OGD/R + HCY + MHY group (Fig. 4d), compared with that in the OGD/R + HCY group. Thus MHY1485 partially reversed the activation effect of Hcy on the autophagic process in OGD/R-injured NSCs.

### ERK activators suppress Hcy-induced autophagy via the ERK-mTOR signaling pathway in NSCs

**Fig. 5:**
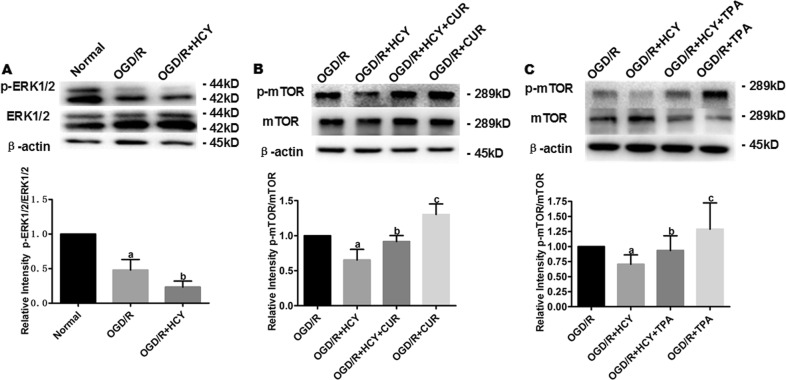
Hcy promotes autophagy in OGD/R-exposed NSCs by inhibiting the ERK-mTOR signaling pathway. **a** Representative western blot for p-ERK1/2. Bar graphs show the semiquantitative levels of p-ERK1/2 as determined by band density analysis. Data from three independent experiments. Note that the quantification of p-ERK1/2 of other groups is normalized to the normal group. Data are expressed as mean ± SD. **a** *P* < 0.05 compared with the normal group. **b** *P* < 0.05 compared with the OGD/R group. **b**, **c** Representative western blot for p-mTOR. Bar graphs show the semiquantitative levels of p-mTOR as determined by band density analysis. Data from three independent experiments. Note that the quantification of p-mTOR of other groups is normalized to the OGD/R group. Data are expressed as mean ± SD. **a**, **c** *P* < 0.05 compared with the OGD/R group. **b** *P* < 0.05 compared with the OGD/R + HCY group

Previous studies have shown that ERK1/2 signaling negatively regulated autophagy induction by activating mTOR, suggesting that inhibition of ERK1/2 could promote autophagy by preventing mTOR activation. Therefore, we hypothesized that Hcy enhances autophagy induction by inhibiting ERK1/2, resulting in mTOR inactivation. Western blot analysis showed that co-treatment with OGD/R and Hcy obviously reduced p-ERK and p-mTOR protein level, compared with that in the OGD/R group (Fig. 5a, *P* < 0.05).

In addition, the effects of activating ERK1/2 on the expression levels of phosphorylated mTOR were also detected in Hcy-treated OGD/R NSCs by western blot. As shown in Fig. 5b, c, ERK1/2 activators (1 µM curcumin for 24 h or 200 nM TPA for 30 min) could significantly enhance the levels of p-mTOR in Hcy and ODG/R co-treated NSCs, compared with that in the ODG/R + HCY group (*P* *<* 0.05). Thus, the effect of Hcy on mTOR was partly mimicked by ERK1/2 activating treatment with TPA or curcumin. It suggested that Hcy may reduce ERK1/2 activity, which inhibits mTOR, leading to the induction of autophagy in NSCs exposed to OGD/R.

### PI3K activator suppresses Hcy-induced autophagy via the PI3K-AKT-mTOR signaling pathway in NSCs

**Fig. 6:**
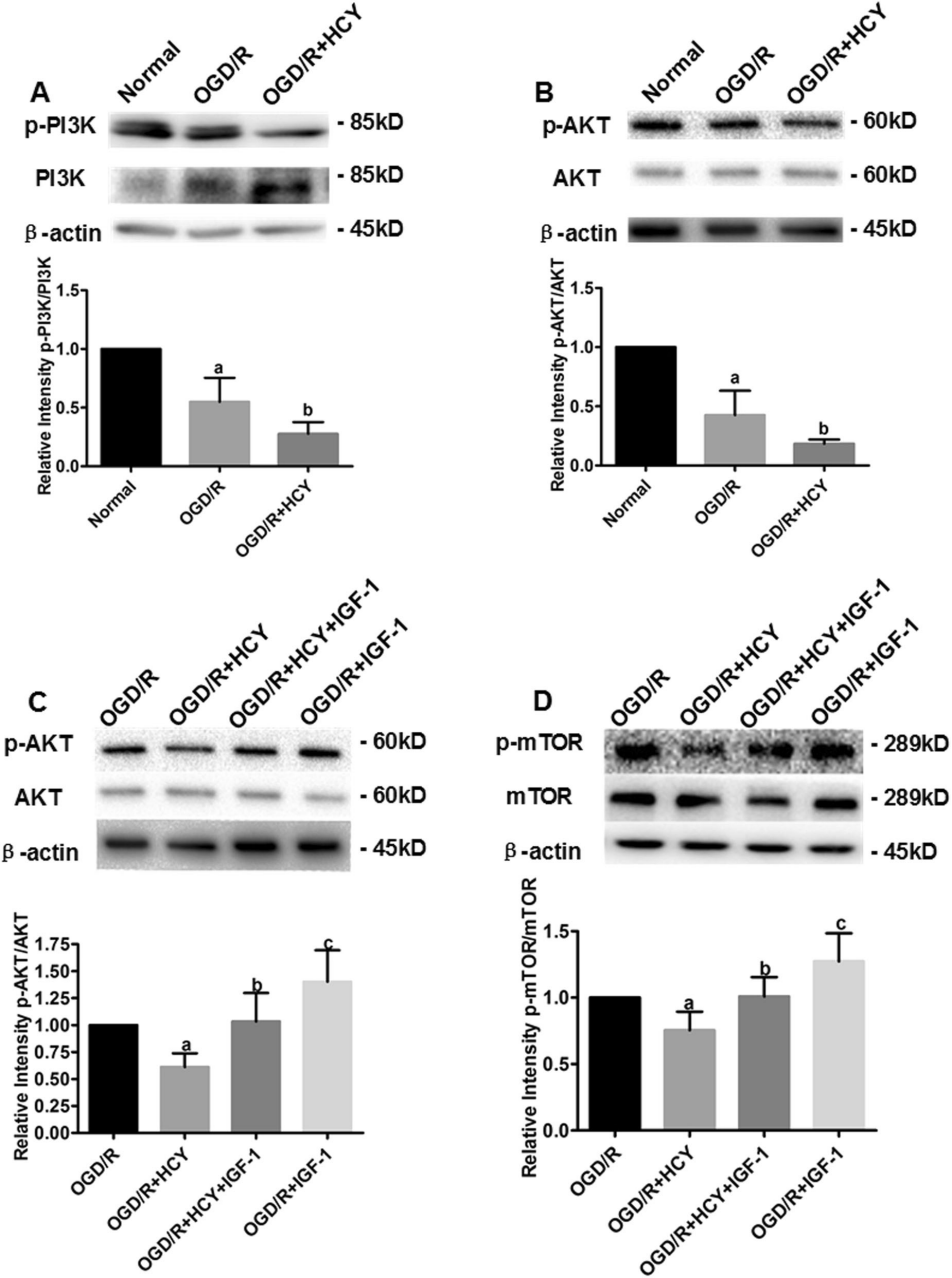
Hcy increases autophagy in NSCs after OGD/R-injury by inhibiting the PI3K-AKT-mTOR signaling pathway. **a**, **b** Representative western blot for p-PI3K and p-AKT, respectively. Bar graphs show semiquantitative levels of p-PI3K and p-AKT as determined by band density analysis. Data from three independent experiments. Note that the quantification of p-PI3K or p-AKT of other groups is normalized to the normal group. Data are expressed as mean ± SD. **a** *P* < 0.05 compared with the normal group. **b** *P* < 0.05 compared with the OGD/R group. **c**, **d** Representative western blot for p-AKT and p-mTOR, respectively. Bar graphs show semiquantitative levels of p-AKT and p-mTOR as determined by band density analysis. Data from three independent experiments. Note that the quantification of p-AKT or p-mTOR of other groups is normalized to the OGD/R group. Data are expressed as mean ± SD. **a**, **c** *P* < 0.05 compared with the OGD/R group. **b** *P* < 0.05 compared with the OGD/R + HCY group

It is generally known that autophagy is negatively regulated by the PI3K-AKT-dependent mTOR signaling in many cell types. Subsequently, the phosphorylation levels of PI3K, AKT, and mTOR in OGD/R NSCs cultured with Hcy were assessed by western blot analysis after the addition of IGF-1 (a selective activator of PI3K) to determine whether the PI3K-AKT-dependent mTOR signaling regulates Hcy-induced autophagy in OGD/R NSCs. As shown in Fig. 6a, b, the level of phosphorylated PI3K and the AKT decreased significantly after OGD/R and Hcy co-treatment. In addition, the significant increase in the levels of p-AKT and p-mTOR were observed after treatment with the PI3K activator IGF-1 (at a concentration of 100 ng/mL for 6 h) in the NSCs exposed to Hcy and OGD/R (Fig. 6c, d). Therefore, addition of the PI3K activator partially abrogated the Hcy-induced suppression of phosphorylation of AKT and mTOR. Hcy enhanced autophagy at least in part via the PI3K-AKT- dependent mTOR signaling pathway in OGD/R-injured NSCs.

## Discussion

**Fig. 7:**
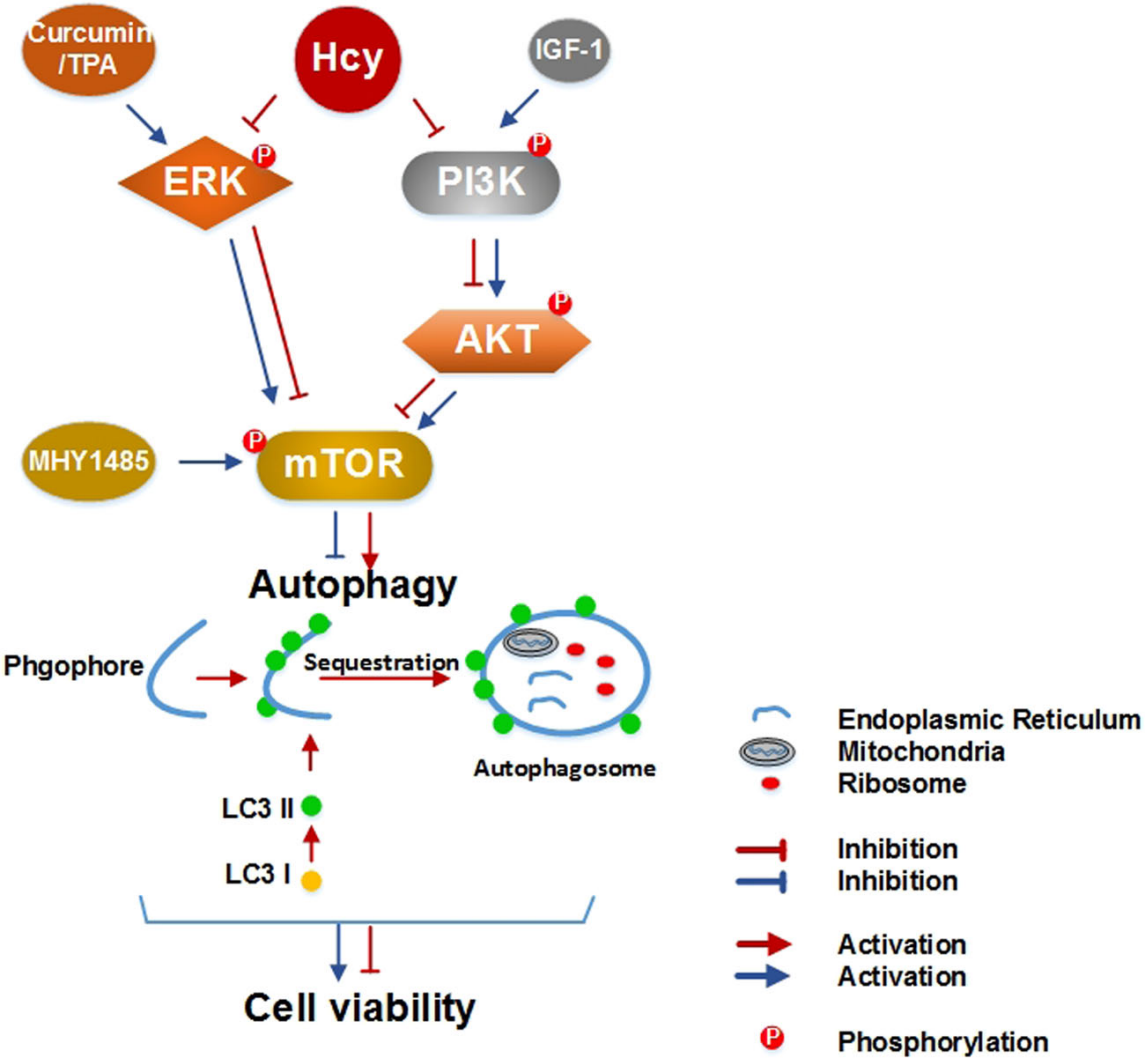
Schematic diagram showing the proposed signaling pathways leading to autophagy activation in the stroke model after Hcy treatment. Hcy exposure causes the inactivation of mTOR, potentially through the ERK and PI3K-AKT signaling pathway, which subsequently leads to autophagy activation. The excessively stimulated autophagy finally contributes to the further decrease in NSCs viability after ischemic injury

The current study provided the first evidence that autophagy of NSCs was sensitive to high Hcy exposure, thereby identifying a novel mechanistic link by which Hcy regulates the cell viability in ischemic brains or OGD/R-injured NSCs. Meanwhile, the ERK1/2 and PI3K-AKT signaling pathways, which co-regulate the phosphorylation of mTOR, play a key role in Hcy-induced autophagy in the NSCs after ischemic injury (Fig. 7).

Epidemiological studies show Hcy, a neurotoxic metabolite, is an independent risk factor for ischemic stroke^5^. However, only limited number of experiments describe the mutual influence of co-morbid high-Hcy level to ischemic damage on cell or animal models of human stroke. Kovalska et al. showed that the combination of high-Hcy level and ischemia reperfusion insult followed by 72 h of reperfusion led to the extensive accumulation of damaged neurons in the CA1 and cortical regions^22^. Jindal et al. found that mild HHcy leads to exacerbation of brain damage and significant deficits in sensory motor function following a transient cerebral ischemia^23^. Our present study provides the direct evidence that Hcy treatment accelerates the progression of OGD/R neural stem cell injury, which corresponds with the findings of previous reported studies. Thus HHcy may be a critical determinant of the severity of ischemic brain damage.

Previous experimental studies from our and other laboratories have documented that the detrimental effect of Hcy after ischemia-induced damage involves the loss of neurons, microglia overactivation, and hypertrophy of astrocytes. For instance, increased neuronal damage by Hcy was detected in in vitro ischemic model of hippocampal slices with OGD^24^. Hcy aggravates cortical neural cell injury through neuronal autophagy overactivation^17^. Hcy exaggerates microglia activation and neuroinflammation through microglia localized STAT3 overactivation following ischemic stroke^25,26^. The present study provides the first line of experimental evidence to demonstrate that the detrimental influence of Hcy may involve negative regulation of NSCs viability through autophagy overactivation. Thus the deleterious effects of Hcy on several major brain cells (neuron, microglia, and NSCs) may contribute to its magnification of the pathogenesis of ischemic stroke.

It has been proven that Hcy has a regulatory role of autophagy in different cell types, however, its regulation displayed two opposite directions (up or down). Khayati et al. found that Hcy activates mTORC1 to inhibit autophagy and form abnormal proteins in human neurons and mice^27^. Similarly, it has been reported that Hcy suppresses autophagy via upregulation endothelin type B receptors in vascular smooth muscle cells^28^. Conversely, Zhao et al. found that Hcy administration enhanced neuronal autophagy, which contributes to cell death following cerebral ischemia^17^. Zhang et al. demonstrated that HHcy-enhanced brain damage is associated with the upregulated autophagy level and neuronal apoptosis in Apo E−/− mice, in which downregulation of Hes1 and Hes5 is involved^29^. Yang et al. reported that Hcy increases autophagy and aggravates liver injury by downregulation of cystic fibrosis transmembrane conductance regulator expression in vivo and in vitro^18^. In this study, we also found that Hcy upregulated autophagy in OGD/R-injured NSCs. Thus, the regulatory effect of Hcy on the level of autophagy varies with the cell type and the biological context.

The in vitro and in vivo studies have highlighted the crucial roles of autophagy-related genes or autophagy regulatory genes in the maintenance of cellular homeostasis and NSCs differentiation into functional neurons. For instance, Yazdankhah et al. suggested that early autophagy regulators, like *Ambra* 1 and *Beclin* 1, on the one hand sustain the stem cell pool within the adult SVZ and, on the other hand, control the level of immature neurons by enhancing the survival of neural precursor cells^30^. Despite the general agreement on the beneficial effects of autophagy activation, excessive autophagy is usually considered to be harmful for the survival and function of NSCs. Li et al. demonstrated that overactivated autophagy in the E14 rats exposed to 3.5% sevoflurane led to increased apoptosis, declined NSC proliferation, decreased relative neuron quantity, and impaired neurocognitive function^31^. More recently it has been reported that OGD strongly induced autophagic cell death in adult rat hippocampal NSCs, and ghrelin treatment protected NSCs from excessive autophagy in experimental stroke^24^. In this study, we also found that the Hcy decreased the viability of rat NSCs by autophagy overactivation by in vivo and in vitro, however, inhibition of autophagy by 3MA protected Hcy-induced NSCs injury. These findings indicated that the role of autophagy may vary with its degree, and the proper level of autophagy is required for NSCs survival.

mTOR signaling is one of the key cellular pathways essential for the regulation of cell growth, survival, and autophagy. It has been shown that the mTOR pathway regulated neural progenitor proliferation in the aging brains^32,33^. For instance, stimulating the mTOR signaling revitalizes the NSCs, restores their proliferation, and enhances neurogenesis in the hippocampus of the aged brain^34^. In addition, there has been growing evidence that the mTOR kinase also responds to some environmental cues to regulate autophagy in NSCs^35^. Yu et al. indicated that the inhibition of mTOR expression was involved in the induction of caspase-dependent apoptosis and autophagy in the methylmercury-exposed human NSCs^13^. Liang et al. showed that Zika virus infection of human fetal NSCs causes inhibition of AKT-mTOR signaling leading to disrupted neurogenesis and aberrant activation of autophagy^14^. Consistent with the findings of previous studies, we found that mTOR-dependent autophagy also mediated the adaptation to Hcy stimuli in OGD/R NSCs. This suggested that the autophagy mediated by mTOR is a key microenvironmental sensor for NSCs survival and differentiation.

It has been shown that PI3K-AKT signal, as the most common upstream targets of mTOR, is essential for neurogenesis from NSCs, as well as for subsequent migration and maturation. In addition, the PI3K-AKT-mTOR pathway was proven to be involved in the regulation of autophagy in NSCs^8^. The inhibitory role of Hcy in the PI3K-AKT signal pathway has been observed in vascular endothelial cells^36,37^. However, the regulation of Hcy on PI3K-AKT-mTOR was not reported in NSCs. Our study reveals for the first time that the inhibition of the PI3K-AKT-mTOR pathway by Hcy treatment led to autophagy overactivation in OGD/R NSCs.

Our previous study proved that Hcy inhibits the proliferation of in vitro isolated NSCs by suppressing ERK signaling^38^. Similarly, it was observed that Hcy exerts an antiproliferative effect on bFGF-stimulated NPCs isolated from the postnatal SVZ, accompanied by inactivation of the ERK^10^. However, it is unclear whether ERK plays the regulatory role of Hcy in NSCs vitality by a direct or indirect way. Here, we showed that Hcy reduced the phosphorylation of ERK and mTOR, and induced autophagy in OGD/R-treated NSCs, and it could be partially reversed by the ERK activators (TPA and curcumin). Although we cannot exclude the possibility that the direct role of ERK in regulating cell vitality, the ERK-dependent mTOR pathway seems to be at least partly responsible for the toxic effect of Hcy on NSCs after ischemic injury.

Taken together, our results demonstrated that combination of high Hcy with ischemic injury induced autophagy overactivation and inhibited cell viability in SVZ NSCs by inhibiting the ERK- and PI3K-AKT- dependent mTOR pathway. This study provides new mechanistic insights on how Hcy exerted neurotoxicity and enhances pathogenesis of stroke, implicating the PI3K-AKT- and ERK- dependent mTOR pathway as potential therapeutic targets for the stroke patient especially due to genetic disorders of Hcy metabolism.

## Materials and methods

### Animals and experimental design

Sixty adult male Sprague-Dawley (SD) rats (160–180 g) (Grade SPF, Certificate Number SCXK (jing) 2016-0006) was purchased from Beijing Vital River Laboratory Co. Ltd (Beijing, China). Use of animals and experimental procedures were conducted in accordance with the Guide for the Care and Use of Laboratory Animals published by the National Institutes of Health (NIH publication no. 80-23, revised 1996). The rats were randomly allocated into five groups: sham operation control group (SHAM), MCAO group, MCAO plus Hcy group (MCAO + HCY), MCAO, Hcy plus 3MA group (MCAO + HCY + 3MA), and MCAO plus 3MA group (MCAO + 3MA). The vehicle and d, l-Hcy (1.6 mg/kg/d; Sigma-Aldrich, St. Louis, MO, USA) were administered by tail vein injection for 28 days prior to SHAM or MCAO operation and up to 7 days after surgery. The concentration of Hcy was chosen based on our previous study^17^, which showed that the treatment of Hcy (1.6 mg/kg/d) for 3 weeks resulted in a notably higher plasma Hcy concentration (15.05 + 2.66 μM) in MCAO rats, compared with nontreated MCAO animals (7.00 + 1.77 μM). The 3MA (5 mM, 4 mL/kg/d) (Sigma-Aldrich) was administered by tail vein injection for 5 days prior to SHAM or MCAO operation.

### Surgical procedures for MCAO

Transient focal ischemia reperfusion was induced by MCAO in the subject animals, and the modified Longa method^39^ was used to assess the neurological deficit. Briefly, the rats were anesthetized by intraperitoneal injection of 1% sodium pentobarbital (40 mg/kg). A midline incision was made at the skin of neck area and the external, internal, and common carotid arteries were carefully exposed. The external carotid artery was tied and the internal carotid artery was closed. A nylon intraluminal suture (length, 18–20 mm, diameter, 0.24 mm) was advanced through the left internal carotid artery to the origin of the middle cerebral artery. One hour after the operation, the suture was slowly and carefully withdrawn to allow reperfusion. Sham-operated controls underwent similar surgical procedures with the exception of suture insertion. The animals were separately euthanized at 3 and 7 days after reperfusion for the subsequent experiments.

### NSCs culture

NSCs were isolated from SD rat SVZ. In brief, the SVZ tissue was dissected out and cut into small pieces, and then digested in trypsin/EDTA solution (0.25% w/v trypsin, 0.02% w/v EDTA, Sigma-Aldrich) at room temperature for 15 min. After filtration through a 200-mesh filter and centrifugation at 300 *g* for 5 min, the cells were cultured in DMEM/F-12 (Sigma-Aldrich) medium containing 20 ng/mL B27, 20 ng/mL epidermal growth factor, and 20 ng/mL bFGF (all from Gibco, Gaithersburg, MD). The cells were maintained at 37 ℃ in a humidified atmosphere under 5% CO_2_. The culture medium was changed every 3 days. All experiments were performed three times in duplicate.

### OGD/R induction and cell treatment

The OGD/R model was established as previously reported^5^. Briefly, cells were cultured in glucose-free medium and then placed in a modular chamber (MC-101 model, Billups-Rothenberg, Del Mar, CA) filled with gas mixture (1% O_2_, 5% CO_2_, and 94% N_2_) at 37 ℃. After 1 h OGD, oxygen–glucose deprivation was terminated by exposing cells at normal culture conditions (37 ℃, 95 % air, and 5 % CO_2_) for 24 h. Cells cultured under normal conditions were used as a normal group.

After purification and culture for 2 days, the cells were treated with 300 μM Hcy throughout the experiment. To determine whether Hcy stimulates the NSCs autophagy, and what are its underlying mechanisms, The NSCs were respectively pretreated with the autophagy inhibitor 3MA (0.05, 0.1, or 0.5 mM) for 72 h, the mTOR activator MHY1485(10 μM) for 24 h, the ERK activator TPA (200 nM) for 30 min and curcumin (1 μM) for 24 h and the PI3K activator IGF-1(100 ng/mL) for 6 h in the presence of Hcy before OGD/R. All experiments were performed three times in duplicate.

### MTT assay and LDH release

Cytotoxicity assessed by the 3-(4,5-dimethylthiazol-2-yl) -2,5-diphenyltetrazolium bromide (MTT) assay and LDH release (all from Nanjing jiancheng, Nanjing, China). The MTT Assay: briefly, the aliquots containing 5 × 10^5^ cells/mL were plated in a 96-well plate. The cells were incubated at 37 °C for 72 h. MTT (0.5 mg/mL) was added to each well at 4 h before the end of the time point, then the MTT reaction was inhibited by addition of 10% SDS and 0.1 M HCl. The formazan crystals formed were dissolved in DMSO, and the absorbance was measured at 490 nm in a microplate reader (Bio-Tek ELX800, Bio-Tek Instrument Inc., Winooski, VT, USA). The LDH assay was performed according to the manufacturer’s guidelines. The percentage of LDH release was calculated by the equation. LDH release (%) = (Experimental LDH release − spontaneous LDH release)/maximum LDH release.

### Transmission electron microscopy and evaluation of autophagy

Cell ultrastructure was observed via TEM. Briefly, the cells were fixed in an isotonic fixative consisting of 4% paraformaldehyde, 2.5% glutaraldehyde, and 0.1 M sucrose in 0.1 M phosphate buffer (pH = 7.4). Fixed monolayers were scraped, then post fixed in the same medium containing 1% osmium tetroxide and subsequently dehydrated in a graded ethanol series (50, 70, 80, 90, and 100%). The dehydrated samples were embedded in epoxy resin and embedded fragments were then sliced and stained with uranyl acetate and lead citrate, and examined under an HT-7700 transmission electron microscope (Hitachi, Tokyo, Japan).

### Western blot analysis

Western blot was performed to analyze protein expression in NSCs. In brief, the cells were homogenized in RIPA buffer (20 mM TRIS-HCl pH 7.5, 150 mM NaCl, 1 mM EDTA, 1% Triton-X 100, 0.5% sodium deoxycholate, 1 mM PMSF, and 10 μg/mL leupeptin), incubated on ice for 30 min, and centrifuged at 14,000 *g* for 10 min at 4 °C. The supernatants were collected for protein concentration detection with BSA (Sigma-Aldrich). Equal amounts of protein from each sample were separated by 12% sodium dodecyl sulfate-polyacrylamide gel electrophoresis and transferred to PVDF membranes (Millipore, Schwalbach, Germany) by wet electrical transfer method. Subsequently, the membranes were blocked with 5% milk (Sigma-Aldrich) in 1 × Tris buffered saline Tween for 1 h at room temperature, followed by incubation with the primary antibodies (rabbit monoclonal anti-LC3 (1:1 000), anti-Beclin 1 (1:1 000), anti-phospho-mTOR (1:1 000), anti-mTOR (1:1 000), anti-phospho-Akt (1:1 000), anti-Akt (1:1 000), anti-phospho-ERK (1:1 000), anti-ERK (1:1 000), anti-phospho-PI3K (1:1 000), anti-PI3K (1:1 000), and rabbit polyclonal anti-caspase-3 (1:1 000), and anti-PARP (1:1 000)) overnight at 4 °C and incubated with the secondary antibody (horseradish peroxidase-linked anti-rabbit IgG, 1:2 000; all from CST, Danvers, MA, USA) for 1 h at 25 °C. The blots were developed by immobilon western chemiluminescent horseradish peroxidase substrate and observed using a ChemiDocTM XRS^+^ Imaging System (Bio-Rad, Hercules, CA, USA). The protein levels were quantified by densitometric analysis using NIH ImageJ 1.61 Software (National Institutes of Health, Bethesda, MD, USA).

### Immunofluorescence staining

Briefly, the rats were anesthetized and perfused with 200 mL 0.9% saline and 4% paraformaldehyde, the brains were removed, fixed in 4% paraformaldehyde and then embedded in paraffin. The sections were dewaxed in xylene (100%) for 15 min, hydrated in a graded alcohol series (100, 90, 75, and 50%) and treated with 3% H_2_O_2_ for 10 min at room temperature. Subsequently the tissues underwent antigen retrieval by incubating the sections for 6 min in a solution of citric acid at 90 °C. The brain sections were blocked with goat serum for 45 min at 37 °C, incubated with the primary antibodies LC3 (1:100; CST, USA) and Nestin (1:50; Sigma-Aldrich) overnight at 4 °C and then incubated with the fluorescein isothiocyanate (FITC)-conjugated goat anti-rabbit or FITC-conjugated goat anti-mouse secondary antibodies (1:50) (zhongshanjinqiao, Bejing, China) for 1 h at 25 °C. 4′,6-diamidino-2-phenylinedole, dihydrochloride (DAPI) (10 μg/mL, Solarbio, Beijing, China) was used to dye the nuclei before 10 min of mounting. The fluorescence images were obtained with an inverted microscope (IX81; Olympus, Tokyo, Japan). LC3 puncta per cell were counted and quantified by Image Pro Plus 6.0 software (Media Cybernetics, Silver Spring, MD, USA).

### Statistical analysis

Statistical analysis was performed using SPSS, version 19.0 (SPSS, Chicago, IL, USA). The results are presented as the means ± standard deviation ($\overline x \pm s$). Differences between means were evaluated by one-way analysis of variance (ANOVA) followed by least significant difference multiple range test. *P* *<* 0.05 was considered to be statistically significant.
